# Automated adaptive detection and reconstruction of quiescent cardiac phases in free-running whole-heart acquisitions using Synchronicity Maps from PHysiological mOtioN In Cine (SYMPHONIC) MRI

**DOI:** 10.1007/s10334-025-01289-5

**Published:** 2025-08-19

**Authors:** Giulia M. C. Rossi Bongiolatti, Nemanja Masala, Jessica A. M. Bastiaansen, Jérôme Yerly, Milan Prša, Tobias Rutz, Estelle Tenisch, Salim Si-Mohamed, Matthias Stuber, Christopher W. Roy

**Affiliations:** 1 Department of Radiology, Lausanne University Hospital (CHUV) and University of Lausanne (UNIL), Rue de Bugnon 46, BH-7-84, 1011 Lausanne, Switzerland; 2 Department of Diagnostic, Interventional and Pediatric Radiology (DIPR), Bern University Hospital, University of Bern, InselspitalLausanne, Switzerland; 3 Translation Imaging Center (TIC), Swiss Institute for Translational and Entrepreneurial Medicine, Bern, Switzerland; 4 Center for Biomedical Imaging (CIBM), Lausanne, Switzerland; 5 Division of Pediatric Cardiology, Woman-Mother-Child Department, Lausanne University Hospital (CHUV) and University of Lausanne (UNIL), Lausanne, Switzerland; 6 Service of Cardiology, Heart and Vessel Department, Lausanne University Hospital (CHUV) and University of Lausanne (UNIL), Lausanne, Switzerland; 7 INSA-Lyon, University of Lyon, University Claude-Bernard Lyon 1, UJM-Saint-Étienne, CNRS, Inserm, CREATIS UMR 5220, U1206, Lyon, France; 8 Department of Radiology, Louis Pradel Hospital, Hospices Civils de Lyon, Lyon, France

**Keywords:** Free-running, Whole-heart, Quiescent cardiac phases, Heart rate variability, Automated detection

## Abstract

**Purpose:**

To reconstruct whole-heart images from free-running acquisitions through automated selection of data acceptance windows (ES: end-systole, MD: mid-diastole, ED: end-diastole) that account for heart rate variability (HRV).

**Methods:**

SYMPHONIC was developed and validated in simulated (*N* = 1000) and volunteer (*N* = 14) data. To validate SYMPHONIC, the position of the detected acceptance windows, total duration, and resulting ventricular volume were compared to the simulated ground truth to establish metrics for temporal error, quiescent interval duration, and volumetric error, respectively. SYMPHONIC MD images and those using manually defined acceptance windows with fixed (MANUAL_FIXED_) or adaptive (MANUAL_ADAPT_) width were compared by measuring vessel sharpness (VS). The impact of HRV was assessed in patients (*N* = 6).

**Results:**

Mean temporal error was larger for MD than for ED and ED in both simulations and volunteers. Mean volumetric errors were comparable. Interval duration differed for ES (*p* = 0.04) and ED (*p* < 10^–3^), but not for MD (*p* = 0.08). In simulations, SYMPHONIC and MANUAL_ADAPT_ provided consistent VS for increasing HRV, while VS decreased for MANUAL_FIXED_. In volunteers, VS differed between MANUAL_ADAPT_ and MANUAL_FIXED_ (*p* < 0.01), but not between SYMPHONIC and MANUAL_ADAPT_ (*p* = 0.03) or MANUAL_FIXED_ (*p* = 0.42).

**Conclusion:**

SYMPHONIC accurately detected quiescent cardiac phases in free-running data and resulted in high-quality whole-heart images despite the presence of HRV.

## Introduction

Whole-heart MRI is increasingly used to assess the cardiac anatomy [1] as it provides three-dimensional (3D) high-resolution images as part of a non-invasive, ionizing radiation-free examination. However, respiratory and cardiac motion, if not adequately addressed, can have a detrimental impact on MR images, resulting in a variety of artifacts (e.g., blurring, ghosting) [2–4] and reduced visibility of small-sized and fast-moving vessels (e.g., coronary arteries) [5]. When accounting for respiratory motion, breath-holds [6, 7] and navigator-gated acquisitions [8–11] can be used, but whole-heart acquisition times generally preclude breath-holding, and irregular breathing patterns can lead to unpredictably long and inefficient acquisitions when using navigators [12]. Alternatively, the advent of free-breathing approaches that either correct [13–19] or resolve [20, 21] respiratory motion can significantly improve scanning efficiency, resulting in shorter and more predictable scan times.

Unfortunately, even sophisticated respiratory motion compensation strategies may result in suboptimal image quality due to residual cardiac motion. In conventional whole-heart imaging, an electrocardiogram (ECG) signal is used to limit the data collection to a quiescent cardiac phase [22] according to a user-defined trigger delay (time from the ECG R-wave) and acceptance window width, which are both based off a CINE pre-scan [23]. A single cardiac phase of interest is typically chosen during mid-diastole or end-systole for coronary imaging [23, 24] but may also be chosen during mid-diastole, end-diastole or end-systole for aortic root measurements [25], or in both using a dual-phase approach for 3D measurements of ventricular function [26]. While often successful in mitigating the effects of cardiac motion, prospective triggering can translate into a time-inefficient, complex, and user-dependent workflow that may be demanding for both the patient and the operator. Furthermore, prospectively chosen trigger delays can lead to missing the cardiac phase of interest if significant variation in the heart rate occurs between the pre-scan and whole-heart acquisition. Additionally, with a fixed acceptance window, variability in the heart rate, ectopic beats, and arrhythmias during the scan cannot easily be accounted for, which may further lead to missing the intended cardiac phase or motion blur [27, 28]. Alternatively, cardiac motion-resolved (CINE) whole-heart techniques [29–31] have been proposed to provide a retrospective assessment of quiescent cardiac phases, thus reducing the possibility of missing the intended cardiac phase. The opportunity for retrospective data selection offers the unprecedented prospect to account for heart rate variability, a characteristic that has, however, not been widely investigated so far. Moreover, reconstruction parameters (e.g., temporal resolution) still need to be defined by the user without a priori knowledge about the position and extent of the rest period, and a manual identification of the rest phase period is still required after an image reconstruction that can take up to several hours. A computationally efficient and automated method for determining motion-consistent data has been proposed for whole-heart imaging [32], but neither does it target a specific cardiac phase, nor does it explicitly account for heart rate variability.

In this work, we, therefore, set out to develop and validate a novel method for reconstructing rest phase whole-heart images from free-running data that automatically selects and adaptively combines data from quiescent cardiac phases while seizing the opportunity offered by free-running acquisitions to account for heart rate variability. The proposed method, Synchronicity Maps from PHysiological mOtioN In Cine (SYMPHONIC) MR, exploits the use of free-running acquisitions [29–31, 33] that continuously sample k-space independent of the underlying physiological motion over a large number of cardiac cycles, ensuring that data from a quiescent cardiac phase is always collected. The data selected by SYMPHONIC are then reconstructed using focused navigation (fNAV) which iteratively reconstructs an image while regionally estimating and correcting for respiratory motion [19]. In this way, motion-consistent whole-heart images of quiescent cardiac phases are obtained in a fixed scan time and without the need for prospective or user-defined acquisition parameters, thus improving scanning efficiency and reducing the expertise required for 3D imaging of the heart.

A comprehensive numerical simulation framework was implemented and used to test two hypotheses. First, that SYMPHONIC can accurately and automatically determine the commonly desired cardiac phases of end-diastole, end-systole, and mid-diastole. Second, that image reconstructions using the automated acceptance windows determined and adapted for each heartbeat by SYMPHONIC are of higher quality than reconstructions of the same data using conventional manually pre-defined acceptance windows with fixed widths. To isolate the effect of accounting for heart rate variability, we further compared our images to reconstructions obtained using the same manually pre-defined acceptance windows but adapted for each heartbeat. Further testing of these hypotheses was performed in a cohort of volunteers and the feasibility of using SYMPHONIC in the presence of high degrees of heart rate variability was explored in a cohort of patients with congenital heart disease (CHD).

## Methods

### SYMPHONIC

**Fig. 1:**
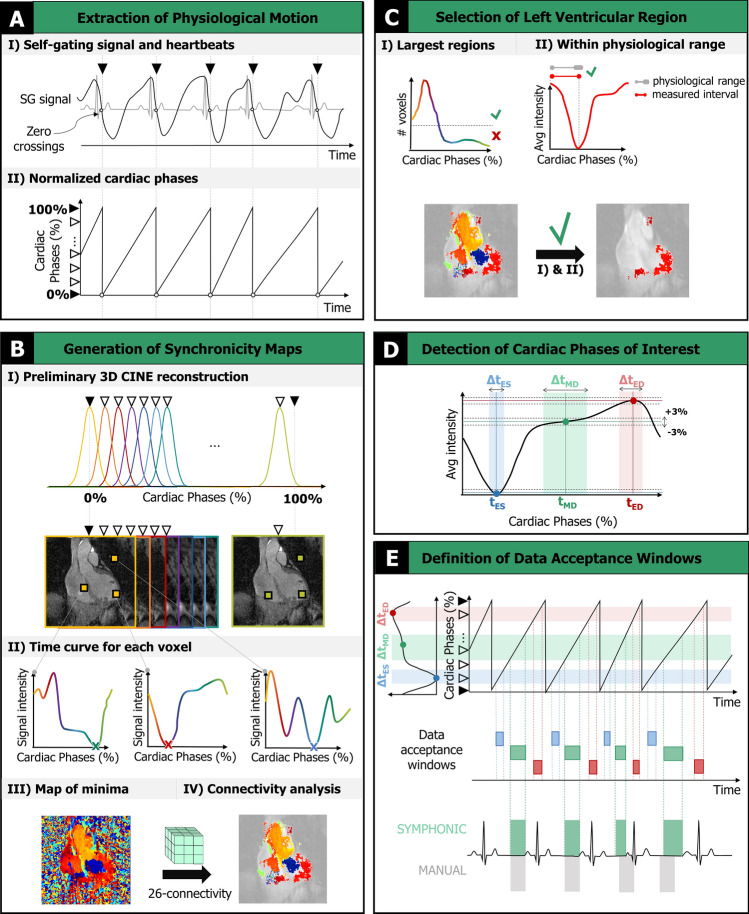
SYMPHONIC algorithm.** A**: Extraction and preparation of physiological motion information. **B**: Generation of a Synchronicity Map displaying connected regions of synchronous voxels. **C**: Refinement of Synchronicity Maps for identification of the left ventricular region. **D**: Detection of timepoints corresponding to cardiac phases of interest (t_ES,_ t_MD_ and t_ED_) and corresponding intervals of quiescent cardiac motion (Δt_ES,_ Δt_ED,_ Δt_MD_). ES: end-systole (*blue*); MD: mid-diastole (*green*); ED: end-diastole (*red*). **E**: Definition of data acceptance windows by mapping the automatically identified quiescent intervals Δt_ES,_ Δt_ED,_ Δt_MD_ back to the original data. Bottom: comparison of adaptive data acceptance windows obtained with SYMPHONIC (*green*) and manually defined acceptance windows with fixed width (*gray*)

SYMPHONIC is used to reconstruct free-running 3D radial data acquired with a spiral phyllotaxis trajectory [34]. The acquisition contains a readout oriented along the superior-inferior (SI) direction for subsequent cardiac self-gating (SG) signal extraction as previously described [30]. Briefly, the spatial information along the SI readouts from each receiver coil is reduced using principal component analysis with two components being designated as cardiac and respiratory SG signals, respectively depending on their power spectra within known ranges for each type of physiological motion [30]. While it is well established that cardiac SG signals exhibit features (i.e., maxima, minima, zero crossing) that can be used to identify individual heartbeats during an MRI acquisition [35], the relationship between the ECG R-wave and such features has not been established so far. SYMPHONIC utilizes cardiac SG signals to automatically identify quiescent cardiac phases and account for heart rate variability in five steps as illustrated in Fig. 1. Following these steps, respiratory motion-corrected rest-phase images can be reconstructed as described in the following sections.

First (Fig. 1A), each heartbeat that occurs during the scan is identified (Fig. 1A-I) by the interval between zero crossings of the cardiac SG signal [30]. To account for heart rate variability, the time stamps of the radial readout lines that coincide with a given heartbeat are mapped to a normalized cardiac phase between 0 and 100% (Fig. 1A-II).

Second (Fig. 1B), a preliminary 3D CINE reconstruction of the data is performed (Fig. 1B-I). Each CINE frame is reconstructed using a NUFFT by applying a Gaussian kernel to all of the acquired data, but centered on the desired cardiac phase (i.e., first frame centered at 0%). A Gaussian kernel width of 5% of the R-R interval is empirically chosen to reduce noise and streaking artifacts. A fixed number of frames (50) are reconstructed to ensure a sufficiently high temporal resolution for subsequent analysis of cardiac motion [36]. Unlike the original self-gating signals which are influenced by motion from different regions of the heart and body, for each spatially separated voxel in these preliminary CINE images, we posit that the signal intensity as a function of cardiac phase (time curve, Fig. 1B-II) contains features related to the underlying cardiac motion, with extrema related to end-diastole (ED) or end-systole (ES), and a plateau related to the mid-diastolic (MD) resting phase. For convenience, we will distinguish the timepoint where the cardiac phase occurs (i.e., t_ED_) from the interval on either side where cardiac motion is quiescent (i.e., Δt_ED_). Unfortunately, the time curves from individual voxels lack the sensitivity to identify specific regions of the heart where periods of quiescent motion can be identified (i.e., left ventricle). To achieve this, a 3D map is generated, where each voxel is associated to the cardiac phase (i.e., one of the fifty frames) at which the minimum of the corresponding time curve is reached (Fig. 1B-III). From this map, spatial connectivity analysis [36] is used to remove noisy or voxels or those from static anatomy and identify the largest regions of the 3D image that exhibit similar motion to their neighbors thus creating a “Synchronicity Map” (Fig. 1B-IV). Connectivity is defined by a 26-connectivity rule [37], meaning that two voxels that reach their minimum in the same cardiac phase are considered connected only if their faces (6 possibilities), edges (12 possibilities), or corners (8 possibilities) are in contact.

Third (Fig. 1C), the Synchronicity Map is further refined by discarding voxels outside the largest connected regions (top 25%, Fig. 1C-I) as well as voxels belonging to regions whose corresponding average time curves yield an interval between extrema (i.e., difference between t_ED_ and t_ES_) that is outside the range of expected physiological values for the given subject (Fig. 1C-II). The range is defined by a parametric model [38, 39] which is a function of the average heart rate of the subject during the acquisition, and where previously reported boundary model parameters for healthy and diseased subjects are used to define acceptance margins. These criteria were chosen based on the observation that the remaining voxels fall within a region containing the left ventricle and can, therefore, be used for subsequent detection of quiescent cardiac phases. In fact, while the first criterion selects the largest regions (e.g., left ventricle, left ventricular outflow tract), the second criterion provides complementary information necessary for the discrimination of the left ventricular region only, where the interval between intensity extrema is expected to match the end-diastolic to end-systolic interval but would not match for the left ventricular outflow tract.

Fourth (Fig. 1D), from the remaining voxels in the Synchronicity Map, the corresponding time curves are averaged, providing the sensitivity needed to identify t_ES_ and t_ED_ based on the minimum and maximum of the averaged time curve, respectively. For t_MD_, a point on the plateau is identified as the midpoint between the first knee point after t_ES_ and t_ED_. The duration of Δt_ED_, Δt_ES_, and Δt_MD_ in terms of cardiac phase is then automatically calculated based on the variation in the time curve at the location of each cardiac phase (empirically chosen range of ± 3% in the amplitude of the average time curve normalized between zero and one). In keeping with standardized CMR protocols [40] the threshold is iteratively lowered until the recommended interval duration of less than 200 ms is reached.

Fifth (Fig. 1E), using the relationship between cardiac phase and the time stamp for each radial readout defined in Step 1, the automatically identified intervals can be mapped back to the original data. As a result, acceptance windows are defined that can be used for subsequent reconstruction of free-running data from numerically simulated and in vivo acquisitions using fNAV for respiratory motion correction [19]. The choice to perform fNAV after the SYMPHONIC algorithm was based on the observation that respiratory motion did not have a significant impact on the synchrony maps (Fig. 1B) nor the resulting signal curves (Fig. 1C) likely due to the avenging of respiratory motion that occurs when reconstructing the preliminary 3D CINE reconstructions (Fig. 1B) and the relative tolerance of radial imaging to motion. Additionally, performing fNAV after SYMPHONIC was more computationally efficient as the fNAV calculations could be performed using only the rest-phase data rather than the entire acquisition.

All algorithms were implemented in Matlab (MathWorks, Natick, Massachusetts, USA). All the reconstructions and analyses described in the following were performed on a workstation equipped with 2 Intel Xeon CPUs (Intel, Santa Clara, California, USA), 512 GB of RAM, and a NVIDIA Tesla GPU (Nvidia, Santa Clara, California, USA).

#### Free-running data acquisition

Free-running data from simulations, volunteers, and patients, was acquired with a 3D radial trajectory with spiral phyllotaxis sampling [34] consisting of 22 segments and 5749 shots (126,478 radial lines), with an isotropic field-of-view of 220 mm^3^ and spatial resolution of 1.15 mm^3^.

##### Numerical simulation data

Free-running numerical simulation data were generated as follows [19]. 3D volumes from the extended cardiac torso (XCAT) phantom [41] spanning fifty phases of a full cardiac cycle were converted to MRI contrast using the analytical equation for a balanced steady state free precession (bSSFP) acquisition [42]. For each time point in the simulated acquisition, the cardiac phase, and the corresponding left ventricular (LV) volume were determined by a model with user defined baseline heart rate (i.e., the average R-wave to R-wave distance, RR), heart rate variability (i.e., the standard deviation of the RR durations, HRV), and a LV volume curve of customizable shape. Then, to generate a given readout, the 3D volume corresponding to the current cardiac phase is chosen and a non-uniform fast Fourier transform (NUFFT) is applied to extract the corresponding k-space line. This process is repeated for all timepoints in the simulated acquisition. The total simulated acquisition followed a 3D radial trajectory with spiral phyllotaxis sampling [31] consisting of 22 segments and 5749 shots (126,478 radial lines), with an isotropic field-of-view of 220 mm^3^ and spatial resolution of 1.15 mm^3^. Note that the simulated free-running datasets did not contain respiratory motion in order to decouple its effects from the subsequent evaluation of SYMPHONIC accuracy and image quality, as described in the following sections.

Simulated datasets (*N* = 1000) were generated across the full range of programmable model parameters as described above, by using a non-linear relationship to model the stretching of cardiac phases with heart rate variability [38]. These included 10 base RR intervals (from 650 to 1100 ms in steps of 50 ms), 10 levels of HRV (from 30 to 165 ms in steps of 15 ms), and 10 different volume curves with realistic variations in the plateau.

##### Volunteer and CHD patient data

Healthy volunteer data (*N* = 14; age: 23–38 years; 10 males) was acquired as part of a previous study [33]. CHD patient data (*N* = 6; age: 10–44 years; 4 males) was retrospectively selected among free-running data routinely acquired at our institution based on criteria of elevated heart rate variability (ratio of HRV over mean RR interval greater than 10%). Acquisitions were performed on a 1.5 T clinical scanner (MAGNETOM Aera and MAGNETOM Sola, Siemens Healthcare, Erlangen, Germany) using a 32-channel spine coil array and an 18-channel chest coil array, while the ECG signal was recorded using a standard 4-lead vector ECG device. All participants, or their legal guardians in the case of minors, provided written informed consent in accordance with our institutional guidelines. Both volunteer and patient data consisted of a 3D radial k-space acquisition with 22 segments and 5749 shots (126,478 radial lines), with an isotropic field-of-view of 220 mm^3^ and spatial resolution of 1.15 mm^3^. All volunteer data were acquired using a previously described free-running 3D radial bSSFP sequence [33], incorporating LIBRE pulses [43–45] for water excitation with a total scan time of 11:20 min, TE/TR: 2.75/5.38 ms, and bandwidth: 1042 Hz/pixel. All patient data were acquired using a previously described free-running 3D radial GRE sequence [46], with a total scan time of 5:59 min, TE/TR: 1.64/2.84 ms, and bandwidth: 1022 Hz/pixel. Prior to scanning, patients received 2 to 5 mg/kg of ferumoxytol (Feraheme, AMAG Pharmaceuticals, Waltham, Massachusetts, USA), injected slowly over 15 min.

#### Validation of cardiac phases detected by SYMPHONIC

##### Numerical simulation data

For each simulated dataset, t_ED_, t_ES_, and t_MD_ (in %) were detected by SYMPHONIC (Fig. 1D) and converted to milliseconds (ms) by multiplying with the average RR interval. The temporal error was then defined as the difference between t_ED_, t_ES_, and t_MD_ (in ms) as detected by SYMPHONIC and the corresponding ground truth values (simulation environment equivalent of ECG). To account for the simulated heart rate variability, the ground truth values of t_ED_, t_ES_, and t_MD_ are based on the known timings mapped to the average RR for a given simulated dataset.

Periods of quiescent cardiac motion can last up to hundreds of milliseconds without significant LV volume changes, which may lead to an increase in temporal error but without impacting image quality. Therefore, for each cardiac phase the volumetric error was additionally computed as the difference between the known simulated LV volume occurring at t_ED_, t_ES_, or t_MD_ as measured by SYMPHONIC and the known average LV volume occurring at ground truth timepoints as detailed above.

##### Volunteer data

In the absence of ground truth values in vivo, preliminary CINE reconstructions from the second step of the SYMPHONIC framework using the recorded ECG signals (Fig. 1B-I) were visually inspected by an expert reviewer (CWR) with ten years’ experience in CMR to manually identify the quiescent intervals Δt_ED_, Δt_ES_, and Δt_MD_ for subsequent comparison to the values obtained by SYMPHONIC. The differences between these parameters when comparing SYMPHONIC and the expert reviewer were tested for statistical significance using paired-sample Wilcoxon signed-rank tests (*n* = 14, *p* <  = 0.05 was considered significant). To demonstrate how the detected quiescent intervals translate in terms of image quality, two representative datasets (with low and high temporal error, respectively) were reconstructed as follows. The quiescent intervals of Δt_ED_, Δt_ES_, and Δt_MD_ detected by both SYMPHONIC and the expert reviewer were converted to data acceptance windows (Fig. 1E) and the selected data were corrected for non-rigid respiratory motion using fNAV [19].

#### Evaluation of SYMPHONIC image quality

##### Numerical simulation data

To assess the impact of SYMPHONIC on image quality all simulated datasets were reconstructed in three ways. First, to emulate conventional whole-heart imaging that uses prospective definitions of acceptance windows with fixed centers and widths, a scout 2D axial CINE dataset was simulated (30 frames, RR interval equal to the mean RR in the first ten seconds of the simulated dataset) and used to visually identify MD and define an acceptance window width. That information was then used to reconstruct a 3D image using a NUFFT, which we refer to as I_MANUAL-FIXED_. Second, the same visually identified interval was used to define an acceptance window with adaptive center and width analogously to the fifth step of SYMPHONIC (Fig. 1E). This information was then used to reconstruct a 3D image using a NUFFT, hereafter referred to as I_MANUAL-ADAPT._ Third, the automatically defined acceptance window with adaptive centers and widths created by SYMPHONIC were used to reconstruct a 3D image using a NUFFT, which we refer to as I_SYMPHONIC_.

Quantitative comparison of I_MANUAL-FIXED,_ I_MANUAL-ADAPT_ and I_SYMPHONIC_ was performed by measuring the right coronary artery (RCA) vessel sharpness (VS) using an automated version of Soap-Bubble that takes into account the known locations of the simulated vessels [19, 47]. Using Soap-Bubble, the percentage vessel sharpness is defined as the ratio between the signal measured along the edge of the vessel and the signal in the center of the vessel. For each simulated combination of RR and HRV, the RCA percentage vessel sharpness for I_MANUAL-FIXED_, I_MANUAL-ADAPT_ and I_SYMPHONIC_ was computed and averaged over the ten different volume curves. The overall differences in VS between the three reconstructions were tested for statistical significance using a paired-sample t-test with Bonferroni correction (*n* = 1000, *p* <  = 0.05 was considered statistically significant).

##### Volunteer data

Healthy volunteer data were also reconstructed in three ways. First, an expert reviewer used a preliminary ECG-gated CINE reconstruction of the free-running data to manually identify MD. The corresponding timepoints were mapped into acceptance windows where, to emulate conventional whole-heart imaging, a window with fixed center and width was used for each heartbeat (I_MANUAL-FIXED_). Second, the same visually identified interval was used to define an acceptance window with adaptive center (I_MANUAL-ADAPT_) and width analogously to the fifth step of SYMPHONIC (Fig. 1E). Third, the automatically defined acceptance window with adaptive center and width created by SYMPHONIC was applied to the data, and I_SYMPHONIC_ was reconstructed also using fNAV. For all three reconstructions resulting acceptance windows were applied to free-running data and reconstructed using fNAV [19] as previously described.

For each given volunteer, the RCA was tracked in each reconstruction (I_MANUAL-FIXED_, I_MANUAL-ADAPT_ and I_SYMPHONIC_), and the VS and visible vessel length (VL) were measured using Soap Bubble (44). The differences in VS between the three reconstructions were tested for statistical significance using paired-sample t-tests with Bonferroni correction (*n* = 14, *p* <  = 0.05 was considered statistically significant).

##### CHD patient data

Each patient dataset was reconstructed as I_MANUAL-FIXED_ and I_SYMPHONIC_ in the same way as described for volunteers. Coronary artery reformats were obtained using Soap Bubble [47] to evaluate the impact of the two reconstruction strategies on detail visibility across the range of baseline heart rates and heart rate variability found in the patient group.

## Results

### Validation of cardiac phases detected by SYMPHONIC

#### Numerical simulation data

**Fig. 2:**
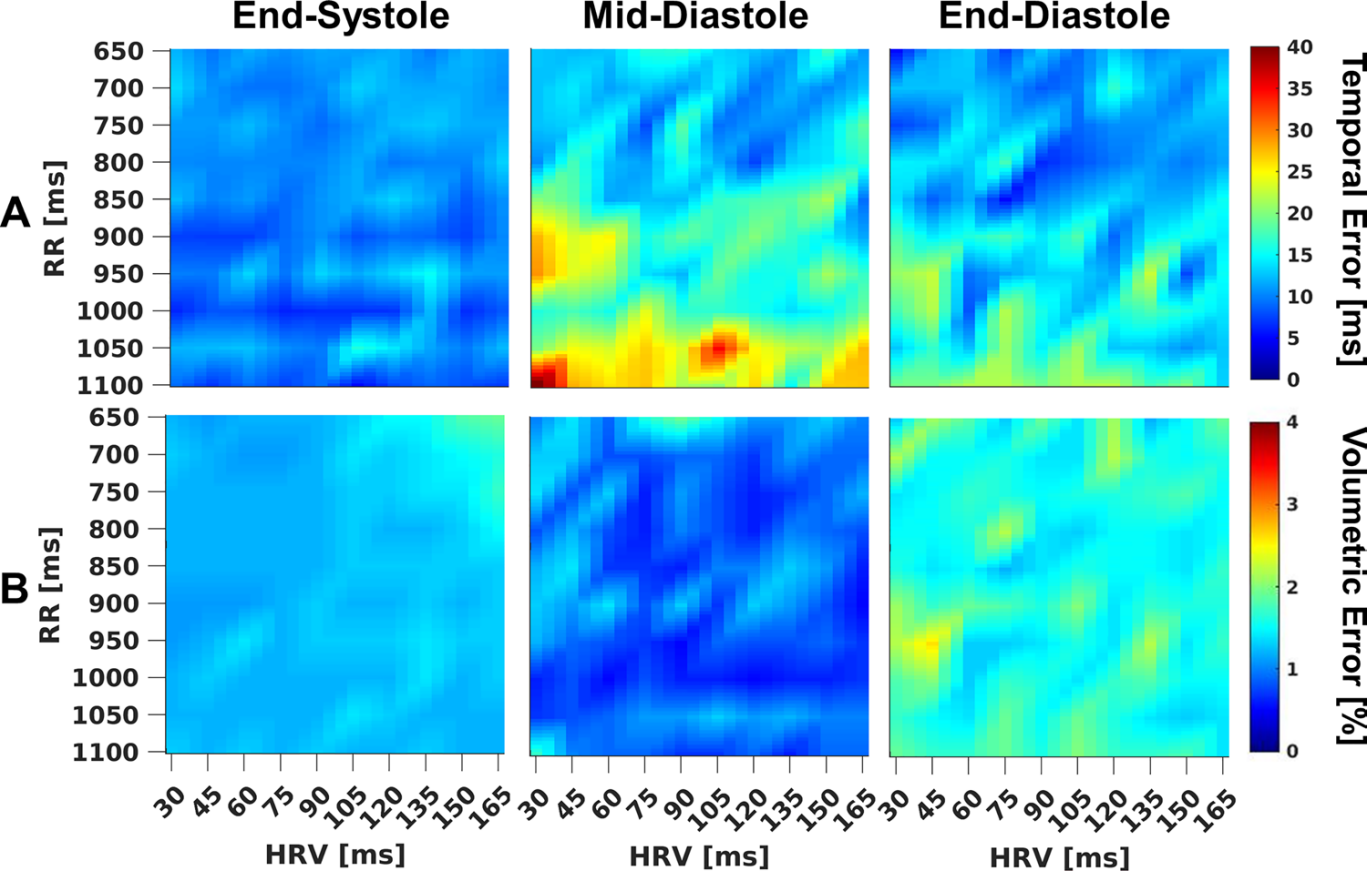
Validation of cardiac phases detected by SYMPHONIC in numerical simulation data. **A** Temporal error (in ms) computed as the difference between cardiac phases (t_ES_, t_MD_, and t_ED_) as detected by SYMPHONIC and corresponding ground values. **B** Volumetric error (in %) computed as the percent difference between the known simulated LV volume occurring at t_ES_, t_MD_, and t_ED_ as measured by SYMPHONIC and the known ground truth LV volume. Errors are reported as the mean absolute errors over the 10 simulated volume curves for end-systole (left), mid-diastole (center) and end-diastole (right)

Cardiac phases detected by SYMPHONIC in simulation data were generally in good agreement with the ground truth (Fig. 2). While t_ES_ was accurately identified with a mean absolute temporal error of 10.3 ± 7.4 ms, the error increased in t_MD_ (17.4 ± 14.1 ms) and t_ED_ (13.7 ± 13.2), especially for long RRs (Fig. 2A)_._ However, these increases in temporal error for t_MD_ and t_ED_ were not reflected in the corresponding volumetric error (Fig. 2B). Independent of the heart rate and its variability, volumetric errors were comparable for the three resting phases (mean volumetric error of 1.6 ± 1.0%, 1.2 ± 0.5% and 0.9 ± 1.0% for t_ED_, t_ES_, and t_MD_, respectively).

#### Volunteer data

**Fig. 3:**
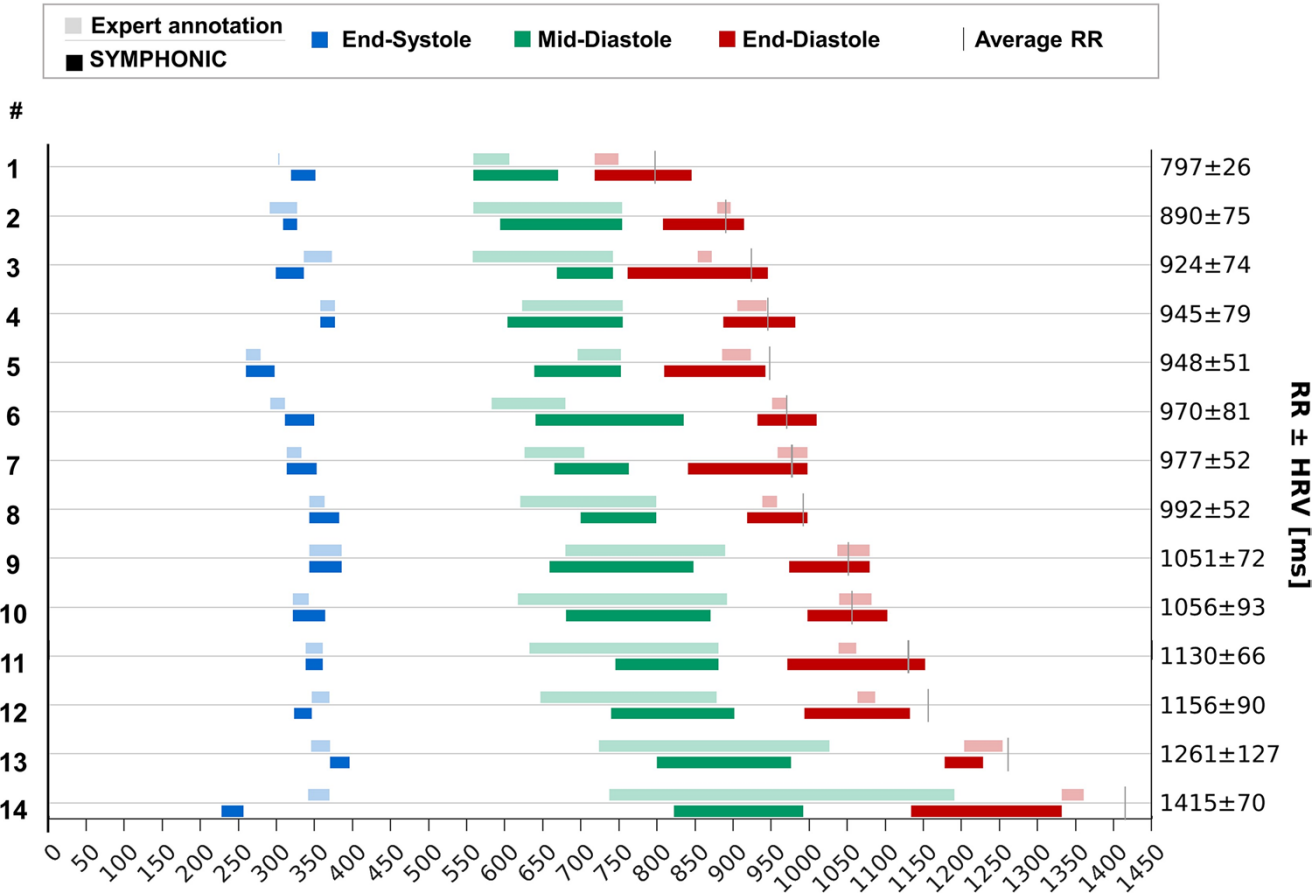
Validation of cardiac phases detected by SYMPHONIC in volunteer data. Quiescent cardiac phases (*blue*: end-systole; *green*: mid-diastole; *red*: end-diastole) detected with SYMPHONIC (*pure colors*) as compared to manual expert annotations (*tinted colors*) in 14 volunteers. The average RR interval (RR) and heart rate variability (HRV) for each volunteer are reported (*y-axis*). For comparability between datasets and despite SYMPHONIC detections being based on self-gating signals, the temporal scale (*x-axis*) is expressed in terms of distance from the ECG R-waves

Cardiac phases detected by SYMPHONIC in volunteer data were in overall good agreement with those visually determined by the expert reviewer (Fig. 3). Again, larger temporal errors were reported for t_MD_ (mean absolute temporal error of 41.0 ± 25.5 ms) as compared to t_ES_ and t_ED_ (mean absolute temporal error of 22.0 ± 29.0 ms and 28.8 ± 28.8 ms, respectively) (Table 1). However, for all cardiac phases, the difference between timepoints was not significant (*p* = 0.64, 0.08 and 0.10 for t_ES_, t_MD_ and t_ED_). In terms of quiescent interval durations, expert detections and SYMPHONIC agreed for Δt_ES_ (23.8 ± 9.8 ms vs 31.8 ± 8.9 ms) and Δt_MD_ (192.3 ± 109.9 ms vs 144.6 ± 39.3 ms), while the Δt_ED_ phase detected by SYMPHONIC was systematically largely exceeding that detected by the expert (124.2 ± 44.0 ms vs 30.8 ± 10.9 ms). The difference in duration was statistically significant for Δt_ES_ (*p* = 0.04) and Δt_ED_ (*p* < 10^–3^), but not for Δt_MD_ (*p* = 0.08).

**Table 1:** Temporal error in volunteer data

#	End-Systole	Mid-Diastole	End-Diastole
	Temporal Error^a^ [ms]	Temporal Error^a^ [ms]	Temporal Error^a^ [ms]
1	31.9	31.9	47.8
2	8.9	17.8	– 26.7
3	– 37.0	55.5	– 9.5
4	0.0	– 9.5	9.5
5	9.5	– 28.5	– 28.6
6	29.1	106.7	9.7
7	9.8	48.8	– 58.6
8	9.9	39.7	10.1
9	0.00	– 31.5	– 31.5
10	10.6	21.1	– 10.6
11	0.0	56.5	11.5
12	– 23.1	57.8	– 11.6
13	25.2	12.6	– 25.0
14	– 113.2	– 56.6	– 113.2
Mean (abs)	**22.0**	**41.0**	**28.8**
Std	29.0	25.5	28.8

^**a**^Temporal error (in ms) computed as the difference between the center point of quiescent intervals identified by SYMPHONIC and the center point of the corresponding expert annotation. Errors for end-systole, mid-diastole and end-diastole are reported for each volunteer separately, as well as in the form of mean absolute errors

**Fig. 4:**
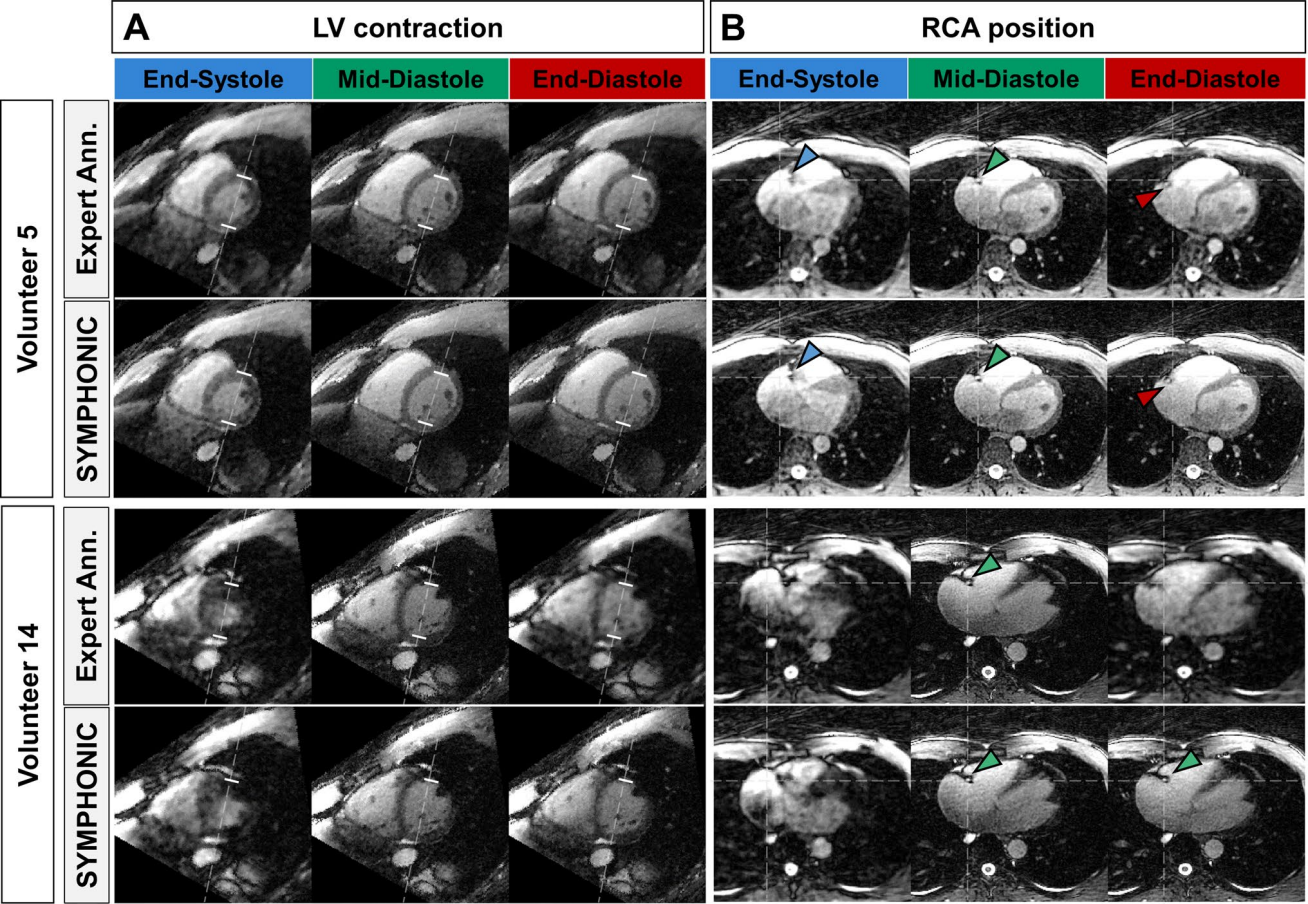
Examples of cardiac phases detected by SYMPHONIC in volunteers from an image perspective. Reconstructions of data within ES, MD and ED acceptance windows obtained with SYMPHONIC are compared to those derived by expert annotations. Volunteer 5 (top) is displayed as a representative case of accurate SYMPHONIC detections (low temporal errors, see Fig. 3). Volunteer 14 (bottom) is displayed as a case of SYMPHONIC misdetections (large temporal errors, see Fig. 3). **A** Short-axis view allowing to visualize the left-ventricular (LV) contraction. *White dashes*: reference LV contraction from expert annotation, mid-diastole. **B** Axial view allowing to visualize the right coronary artery (RCA) groove. *White dashed lines*: reference RCA position from expert annotation, mid-diastole. *Arrows*: end-systolic (*blue*), mid-diastolic (*green*) and end-diastolic (*red*) RCA position

For Volunteer 5 (representative case of accurate SYMPHONIC detections, with temporal error of 9.5 ms, − 28.5 ms and − 28.6 for t_ES_, t_ED_ and t_MD_) images reconstructed from data within Δt_ES_, Δt_ED_, and Δt_MD_ as identified by either SYMPHONIC or the expert reviewer were in good agreement as far as the LV contractile state (Fig. 4A, *top*) and the RCA position (Fig. 4B, *top*) are concerned. For Volunteer 14 (case of SYMPHONIC misdetection, with temporal errors − 113.2 ms, − 56.6 and − 133.2 ms for t_ES_, t_MD_ and t_ED_), images reconstructed from data within Δt_MD_ as detected by SYMPHONIC and the expert reviewer were also comparable both in terms of LV contractile state (Fig. 4A, *bottom*) and RCA position (Fig. 4B, *bottom*). However, the impact of misdetection was evident in images corresponding to data from Δt_ED_ as detected by SYMPHONIC, where the RCA position was closer to mid-diastole than to end-diastole (Fig. 4B, *bottom*). For both volunteers, images reconstructed from data during Δt_MD_ and Δt_ED_ were comparable to images reconstructed from data within Δt_MD_ and Δt_ED_ defined by expert annotations, while all images reconstructed from data within Δt_ES_ contained visible undersampling artefacts.

### Evaluation of SYMPHONIC image quality

#### Numerical simulation data

**Fig. 5:**
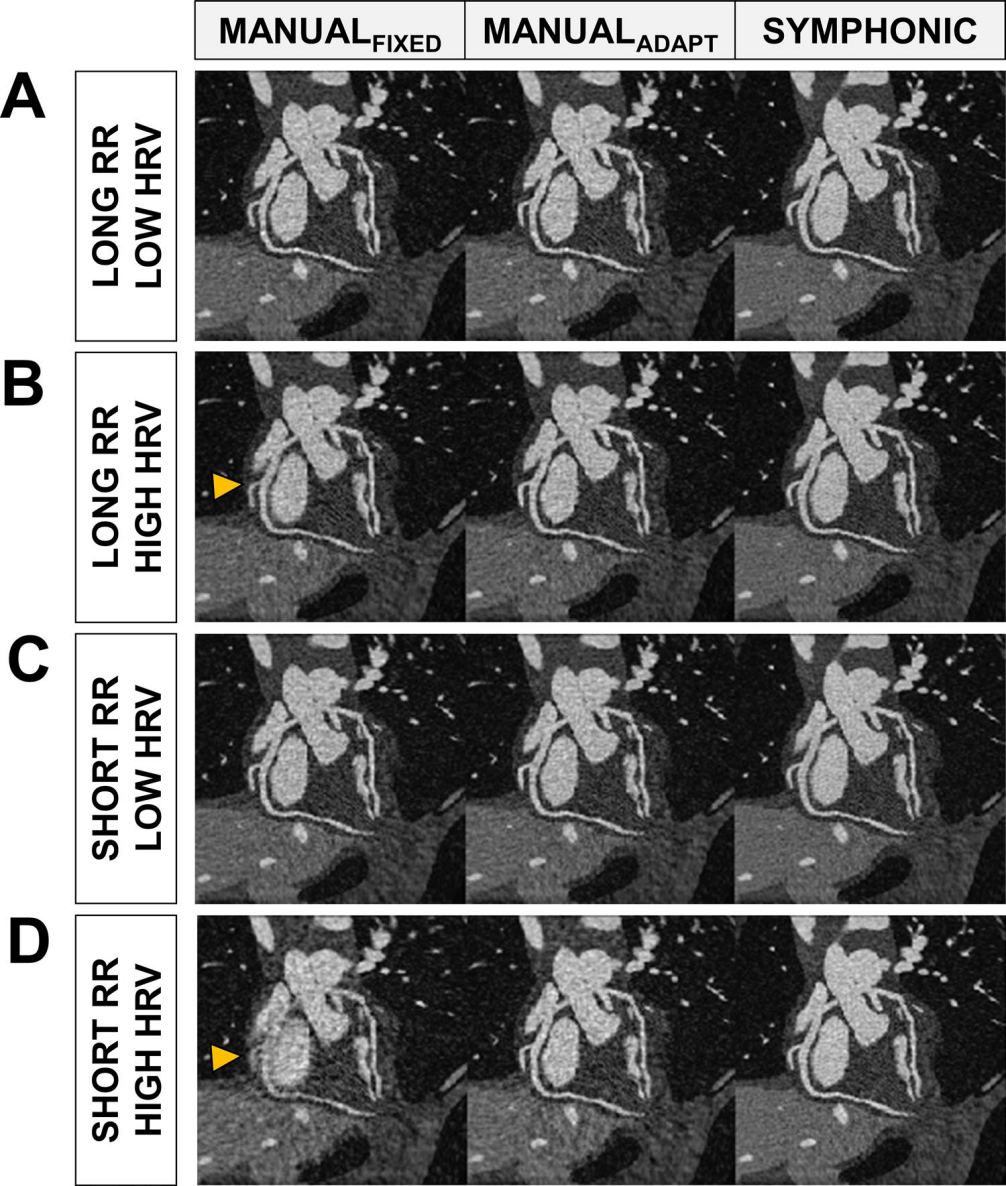
Examples of I_MANUAL-FIXED,_ I_MANUAL-ADAPT_ and I_SYMPHONIC_ image quality under different simulated heart rate (RR) and heart rate variability (HRV) conditions in numerical simulation data. Reformatted images of the right coronary artery (RCA) and left anterior descending coronary artery (LAD) obtained from I_MANUAL-FIXED,_ I_MANUAL-ADAPT_ and I_SYMPHONIC_ reconstructions of four representative datasets simulated with different combinations of RR (High: 1100ms; Low: 650ms) and HRV (High: 165ms; Low: 30ms). *Yellow arrows*: example locations where SYMPHONIC allows for higher-quality visualization of coronary arteries

For each simulated dataset, the quiescent 3D volumes I_SYMPHONIC_ and I_MANUAL-ADAPT_ and I_MANUAL-FIXED_ were qualitatively similar (Fig. 5). In terms of motion blur, the shorter the RR duration and the higher the HRV, the more significant were the visual improvements brought by I_SYMPHONIC_ and I_MANUAL-ADAPT_ over I_MANUAL-FIXED_, with I_SYMPHONIC_ and I _MANUAL-ADAPT_ providing improved visualization of vessels in the corresponding RCA and LAD reformats, even in extreme HRV cases (Fig. 5D).

**Fig. 6:**
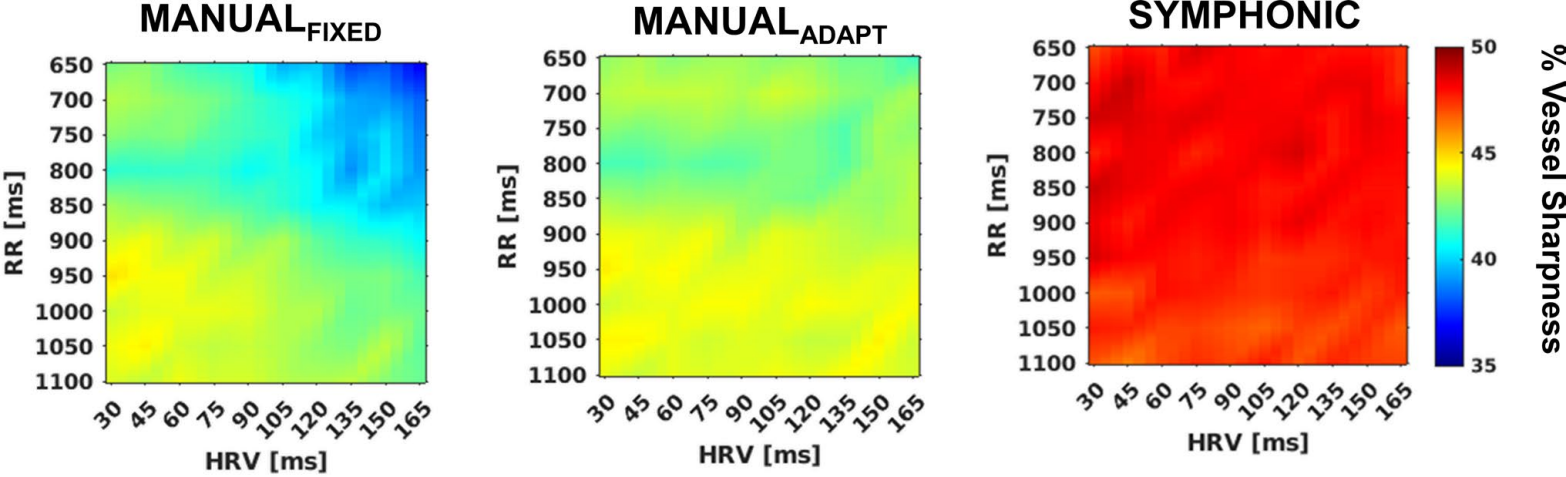
Quantitative evaluation of I_MANUAL-FIXED_, I_MANUAL-ADAPT_ and I_SYMPHONIC_ image quality under different heart rate (RR) and heart rate variability (HRV) conditions in numerical simulation data. Right coronary artery (RCA) percentage vessel sharpness (VS) measurements obtained from I_MANUAL-FIXED_ (left), I_MANUAL-ADAPT_ (center), and I_SYMPHONIC_ (right) reconstructions of datasets simulated with different combinations of RR (from 650 to 1100 in steps of 50ms) and HRV (from 30 to 165 in steps of 15). Each point represents the mean across the 10 simulated volume curves

These qualitative results were corroborated by quantitative RCA percentage vessel sharpness (VS) measurements across the 1000 simulated datasets for the three reconstruction methods. I_SYMPHONIC_ provided a consistent VS close to 50% for the entire evaluated RR and HRV range. Similarly, I_MANUAL-ADAPT_ provided a consistent VS throughout the simulated ranges, while for I_MANUAL-FIXED_, increasingly higher HRV and shorter RR intervals led to a progressively reduced vessel sharpness (Fig. 6). For both I_MANUAL-ADAPT_ and I_MANUAL-FIXED_, the VS was always lower than that from I_SYMPHONIC_. The observed difference between I_SYMPHONIC,_ I_MANUAL-ADAPT_ and I_MANUAL-FIXED_ were statistically significant (47.8 ± 1.9 vs 43.3 ± 1.4 vs 42.0 ± 2.4, *p* < 0.001).

#### Volunteer data

**Fig. 7:**
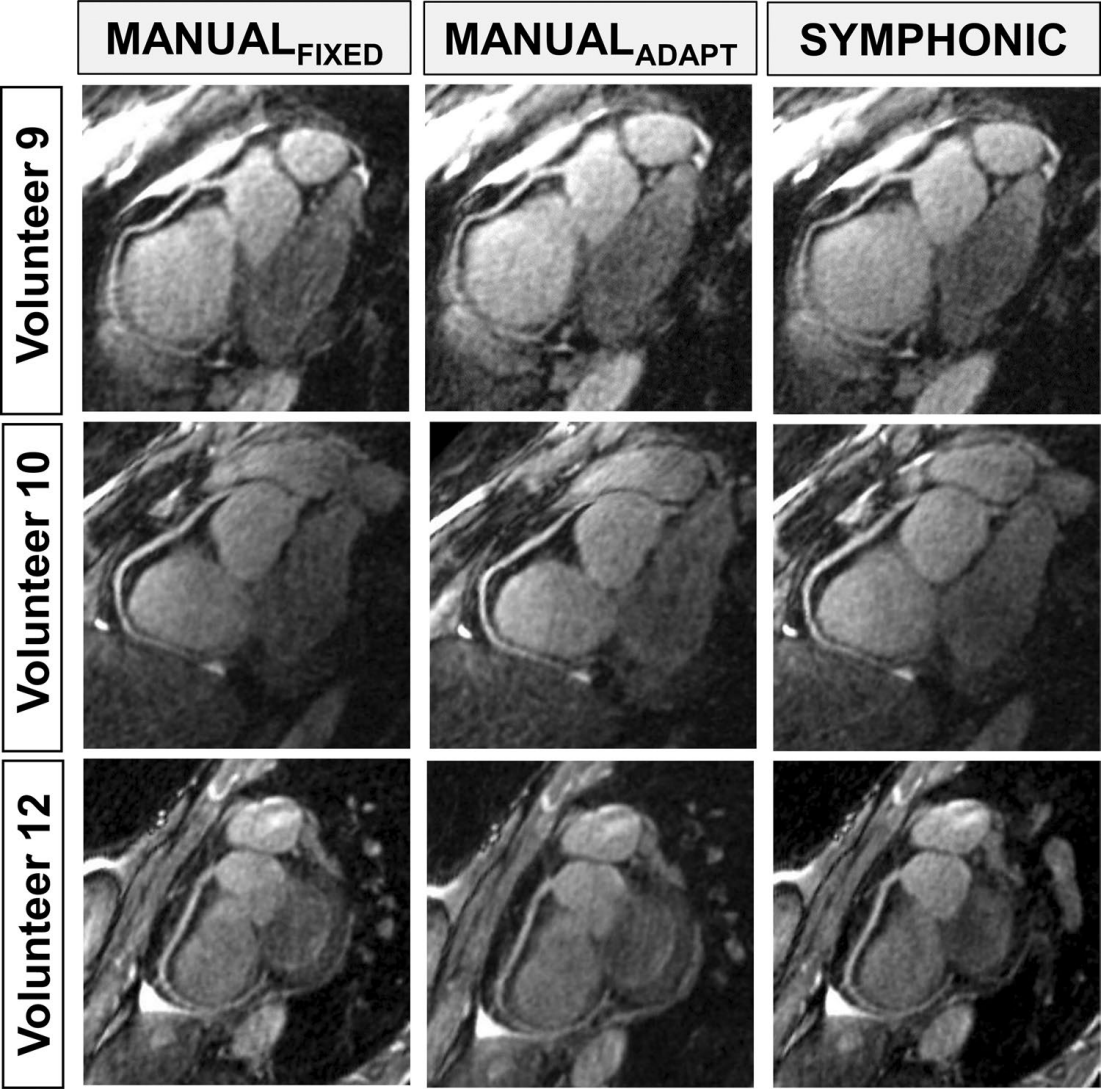
Examples of RCA reformats issued from I_MANUAL-FIXED_, I_MANUAL-ADAPT_ and I_SYMPHONIC_ in volunteer data. RCA reformats for three different volunteers are shown

For each volunteer, the image quality of I_SYMPHONIC,_ I_MANUAL-ADAPT_ and I_MANUAL-FIXED_ was visually similar. The resulting RCA reformats were also visually comparable in terms of image quality and vessel conspicuity in the majority of cases (Fig. 7).

**Fig. 8:**
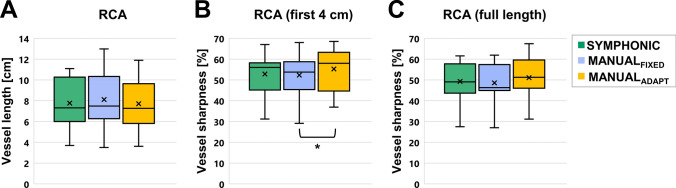
Quantitative evaluation of I_MANUAL-FIXED_, I_MANUAL-ADAPT_ and I_SYMPHONIC_ image quality in volunteer data. **A:** RCA vessel length; **B:** RCA percentage vessel sharpness over the first four centimeters; **C:** RCA percentage vessel sharpness over the full tracked length. * Statistically significant differences. *Cross*: mean; *Central bar*: median

Quantitative indicators of image quality (Fig. 8) showed a mean vessel sharpness (VS(4 cm) and VS(full length)) of 52.9 ± 10.0% and 49.3 ± 9.6% for I_SYMPHONIC_, which was very close to that from I_MANUAL-FIXED_ (52.3 ± 10.1% and 48.6 ± 9.4%) and I_MANUAL-ADAPT_ (55.5 ± 10.3% and 51.4 ± 9.5%).A statistically significant difference was found between I_MANUAL-FIXED_ and I_MANUAL-ADAPT_ for the VS(4 cm) (*p* < 0.01), while none of the other differences were statistically significant.

#### CHD patient data

**Fig. 9:**
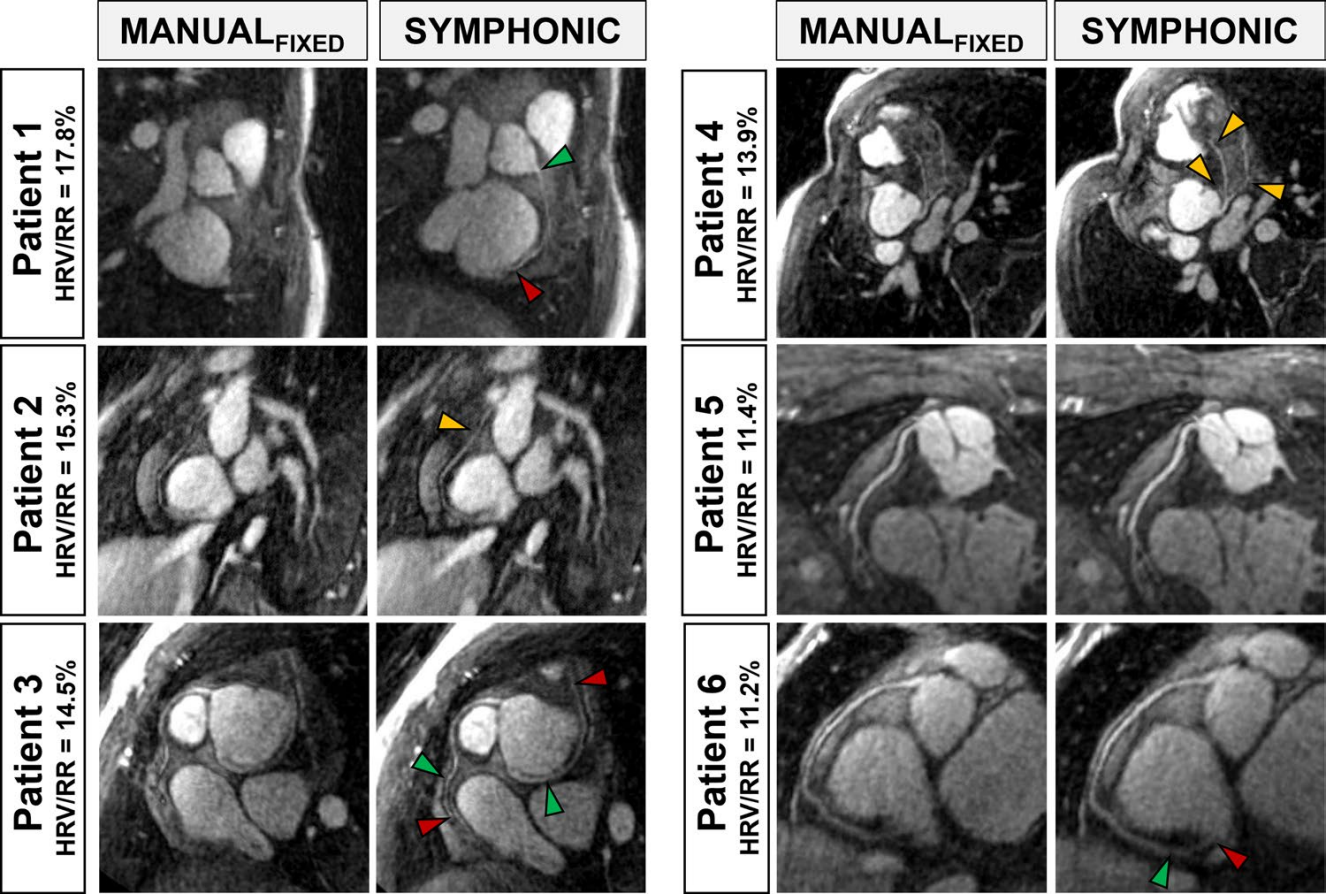
Examples of coronary reformats issued from I_MANUAL-FIXED_ and I_SYMPHONIC_ for congenital heart disease patients with high HRV. Arrows indicate locations (*yellow*) and portions (*green*: start, *red*: stop) where SYMPHONIC allows for a better depiction of coronary arteries

In patients with high heart rate variability (HRV/RR > 10%), I_SYMPHONIC_ provided a clear improvement in the conspicuity of fine anatomical structures when compared to I_MANUAL-FIXED_. Coronary artery reformats obtained from patients with increasingly high heart rate variability (HRV/RR from 11.2% to 17.8%) further demonstrate that I_SYMPHONIC_ provides a better depiction of fine anatomical details when compared to I_MANUAL_ (Fig. 9).

## Discussion

SYMPHONIC, a novel method for the extraction of motion-consistent whole-heart images from free-running data that accounts for heart rate variability was developed and validated. A simulation framework was developed to provide known ground truth under a wide range of conditions that mimic physiological variability. Using simulation and volunteer data, we successfully tested the hypothesis that SYMPHONIC based on cardiac self-gating can accurately and automatically determine the cardiac phases of end-diastole, end-systole, and mid-diastole with validation against conventional ECG-gating. Then, using simulation, volunteer, and patient data, we successfully tested the hypothesis that images reconstructed using SYMPHONIC demonstrate overall improved quality when compared to reconstructions of the same data using the conventional approach of manually defined acceptance windows and fixed window widths. An additional comparison with images obtained using the same manually defined acceptance windows but adapted for each heartbeat allowed us to assess for the first time the benefits of accounting for heart rate variability in free-running acquisitions.

Overall, quiescent cardiac phases detected by SYMPHONIC were in good agreement with the ground truth in simulations and with those identified by an expert reviewer in volunteer data. The temporal detection errors were larger for t_MD_ than for t_ES_ and t_ED_. However, in simulations, the volumetric error was consistent between the three cardiac phases. This suggests that the increase in temporal error is due to a longer cardiac resting phase, in which the volume of the ventricle increases more gradually with longer RR and in turn should have less impact on image quality.

In simulations, SYMPHONIC proved effective in providing consistent image quality across a large range of HRV/RR. This consistency is to be attributed to the ability of SYMPHONIC to account for heart rate variability, as it was also observed in reference reconstructions that similarly used acceptance windows with adaptive centers and widths. Conversely, in reference reconstructions that did not account for heart rate variability image quality progressively degraded as HRV/RR increased. No significant improvement in image quality attributable to SYMPHONIC was found in the volunteer study, where only low HRV/RR levels (< 10%) were represented, and where sharpness measurements might have been affected by additional factors (i.e., noise, uncorrected respiratory or bulk motion) that were not necessarily accounted for in the simulation. Nonetheless, in CHD patients, SYMPHONIC offered improved detail definition with high HRV/RR (> 10%) as compared to reconstructions that do not account for heart rate variability.

SYMPHONIC builds on previous work using free-running acquisitions [30] to overcome the potential limitations of prospective ECG-triggering. The continuous nature of the free-running acquisition offers the opportunity to retrospectively identify suitable windows for anatomical visualization while retrospectively accounting for variability in physiological motion. This is not easily achievable with conventional ECG-triggered approaches that require prospective, manual selection of data acceptance windows with fixed widths. The impact of heart rate variability in prospective and retrospective approaches has not been widely investigated previously, and our work thus constitutes an initial assessment of the added value of methods able to tackle it.

In published reconstructions of free-running data [30], the need for compressed sensing results in long reconstruction times (on the order of several hours, requires a significant amount of RAM (~ 200 GB) in order to produce cardiac and respiratory resolved 5D images, and quiescent cardiac phases still need to be manually identified a posteriori. SYMPHONIC significantly reduces the reconstruction time (e.g., 22 ± 4 min for the obtention of mid-diastolic images from volunteer data) as well as the computational demands (~ 20 GB of RAM), and the fully automated process additionally promotes operator independency. Nevertheless, if 5D images are desired, SYMPHONIC could be combined with a 5D reconstruction pipeline to adapt to heart-rate variability and automatically identify rest phases in the resulting 5D reconstructions. As opposed to the previously published method for automated extraction of motion-consistent data from free-running acquisitions [32], SYMPHONIC allows for the extraction of specific physiological cardiac phases, thus ensuring the obtention of the desired image and further promoting consistency. Furthermore, the amount of data included in SYMPHONIC MD acceptance windows in concert with the integration of fNAV, resulted in high quality images in a scan time that is consistent with other methods for whole-heart imaging (volunteers: 11:33 min [33], patients: 5:59 min [46]). It is therefore natural to further explore additional acceleration to determine the under-sampling limit for SYMPHONIC and by extension the total acquisition time needed according to the underlying free-running sequence as was recently demonstrated for ferumoxytol-enhanced free running imaging [49]. However, for higher levels of undersampling compressed sensing of the single 3D frames may be required which will incur additional computational time and then merit a comparison to previously established methods for cardiac and respiratory motion-resolved whole-heart 5D imaging which relies on compressed sensing [30, 31]*.*

While previous methods aimed at identifying cardiac phases from CINE images do exist, those were mainly developed with the aim of automatizing the selection of the acquisition window for ECG-triggered imaging, and therefore, focused on detecting periods of cardiac quiescence from 2D CINE images obtained at the pre-scan stage from an amalgam of multiple heartbeats. In this context, a variety of approaches have been proposed that use traditional signal or image processing techniques to identify cardiac resting phases [50–52], anatomical landmark detection and cardiac motion tracking [53] or deep learning algorithms [54]. Our approach lays in-between the first two categories, as cardiac motion is simply derived by processing temporal signals, which are nonetheless obtained from an automatically determined specific anatomical portion.

In the current study, SYMPHONIC was developed and applied to 3D radial data. However, the concept could be translated to other free-running trajectories, including Cartesian [55, 56] and spiral acquisitions [57]. Free-running Cartesian data may benefit more significantly from SYMPHONIC as the presence of heart rate variability often leads to more coherent artifact and blur than in non-Cartesian methods. Additionally, the current approach used cardiac self-gating signals to reconstruct the initial CINE needed for cardiac phase detection, but SYMPHONIC can be readily extended to use an ECG or pulse oximetry signal recorded in parallel to the acquisition or combined with the pilot-tone navigation system [58, 59].

In this work, we chose to reconstruct ED, ES, and MD, in keeping with common use cases in clinical imaging. However, one cardiac phase may suffice for specific applications including coronary imaging and aortic root measurements, while two phases may be needed for ventricular function measurements. Further study is warranted to compare these specific use cases to established prospectively ECG-triggered imaging and ECG-gated CINE imaging to determine the diagnostic utility of SYMPHONIC images and evaluate whether the multiple phases that SYMPHONIC can simultaneously detect and reconstruct provide additional value.

### Limitations

The simulated MRI physics for numerical data does not consider sources of artifacts such as off-resonance, field-inhomogeneity, and flow-related dephasing. This may account for the discrepancies in the temporal errors calculated from simulated and volunteer data. This may be particularly true for the outlier case of Volunteer 14, where flow-related signal dephasing in the aorta misled the algorithm to select pixels in the aortic blood pool rather than the LV. This resulted in large temporal errors corresponding to the observed timing between the moment of maximal signal dephasing in the aorta and ES. However, owing to the long RR, the mid-diastolic images were still in good agreement with reference mid-diastolic images, as anticipated by simulation results showing that temporal detection errors do not directly translate into volumetric differences when the RR is sufficiently long. As a result of the shortcomings of the simulation, further validation was performed in volunteers where we exploited the recorded ECG to create reconstruction that are similar to conventional ECG-triggered images. Further study is warranted comparing SYMPHONIC images to separately acquired prospectively ECG-triggered images, which benefit from magnetization-recovery in-between heartbeats. Additionally, such a study in a larger patient cohort would provide insight into the occurrence rates for high levels of heart rate variability and their true impact on ECG-triggered acquisitions.

To account for heart rate variability in vivo, the cardiac cycles were normalized to a uniform length (Fig. 1A-II) and this approach appears to work well for the quiescent phases reconstructed in this study (ED, ES, and MD). Nevertheless, it is established that systole and diastole do not stretch linearly with cardiac cycle length [60] and therefore, the current linear approximation may be improved upon. However, this may require further patient-specific tuning to account for coefficients used in non-linear models of cardiac cycle lengths [60]. It should be noted that any normalization strategy can be valid only for standard heartbeats that comply with the model and if arrhythmic beats occur intermittently to normal heart beats (e.g., ectopic beats such as premature atrial or ventricular contractions), implementing an arrhythmia rejection or classification strategy [61, 62] may help improve images reconstructed with SYMPHONIC. In such a case, additional information from a recorded ECG (i.e. P or T waves) may be useful to stratify the ectopic heartbeats although it would be interesting to see how such beat impact the temporal curves shown in Fig. 1B leaving the door open for SYMPHONIC to be modified to detect arrhythmias.

The empirically chosen parameters of SYMPHONIC, require further study in a larger patient cohort to establish their robustness. In particular, in our work the automatic detection of acceptance window widths was based on a threshold on the variation of the time curve that was defined empirically based on the observation that, for real volume curves obtained from manual segmentations, the volume variation during the mid-diastolic plateau falls within that range. The threshold was, however, not adapted, and may therefore be further optimized, for end-systole and end-diastole, which may explain the mismatch in duration between ES and ED intervals detected by SYMPHONIC and those annotated by the expert. Additionally, it should be noted that our conservative threshold choice leads to an unpredictable amount of data included in the reconstruction. As a result, the noise characteristics and aliasing in the resulting images may vary and SYMPHONIC may not exploit redundancies in free-running data as well as previously published 5D image reconstruction techniques. This could potentially be addressed by adding a constraint on the minimal amount of data to be included, albeit with the potential for motion-blur if the heart rate is high unless mitigated by a method for intra-cardiac motion correction [63].

## Conclusion

SYMPHONIC, a novel fully automated algorithm for retrospective selection of periods of cardiac quiescence in free-running data that adaptively accounts for heart rate variability without the need for user interaction, was developed and validated. The proposed method provides an accurate and automated detection of clinically relevant cardiac phases and the reconstruction of high-quality whole-heart images despite the presence of significant heart rate variability. Consequently, the use of SYMPHONIC may be particularly beneficial in patients with fast heart rates, arrhythmias, drifts in the baseline heart rate or significant beat-to-beat changes in the duration of the cardiac cycle.

## Data Availability

The datasets and algorithms used and analyzed during the current study are available from the corresponding author upon reasonable request.
